# Adaptive behaviour during epidemics: a social risk appraisal approach to modelling dynamics

**DOI:** 10.1098/rsif.2024.0363

**Published:** 2025-01-15

**Authors:** David O’Gara, Matt Kasman, Laurent Hébert-Dufresne, Ross A. Hammond

**Affiliations:** ^1^ Division of Computational and Data Sciences, Washington University in St Louis, One Brookings Drive, St Louis, MO 63105, USA; ^2^ Center on Social Dynamics and Policy, Brookings Institution, 1775 Massachusetts Avenue NW, Washington, DC 20036, USA; ^3^ Vermont Complex Systems Center, University of Vermont, 82 University Place, Burlington, VT 05405, USA; ^4^ Department of Computer Science, University of Vermont, 82 University Place, Burlington, VT 05405, USA; ^5^ Brown School, Washington University in St Louis, One Brookings Drive, St Louis, MO 63105, USA; ^6^ Santa Fe Institute, 1399 Hyde Park Road, Santa Fe, NM 87501, USA

**Keywords:** contagion, computational epidemiology, behavioural response

## Abstract

The interaction of infectious diseases and behavioural responses to them has been the subject of widespread study. However, limited attention has been given to how broader social context shapes behavioural response. In this work, we propose a novel framework which combines two well-studied dynamic processes into a ‘social risk appraisal’ mechanism. Our proposed framework has both theoretical and empirical support, occupying an important middle ground in the interacting contagions literature. Results indicate that a risk appraisal framework can express a wide range of epidemic outcomes, driven by simple interaction rules. This framework has implications for designing containment strategies in disease outbreaks, as well as equity considerations. Finally, the risk appraisal approach is well-posed to engage with a broad set of literature in epidemic management, decision-making and the adoption of social behaviours.

## 1. Introduction

The dynamics of infectious disease spread in humans are not solely dependent on the pathogen at hand and the pattern of contact present at the beginning of an epidemic. Decades of theoretical and empirical work have demonstrated how social and behavioural factors can mitigate or amplify disease outbreaks, in large part because of their own spread within social systems [1]. In mathematical models, and especially in individual (or ‘agent-based’) simulations, the mechanism of contagion has proved an invaluable tool to operationalize both the transmission of infectious disease and the adoption of social behaviour [2–4]. To date, most work has modelled adaptive behavioural spread via either complex contagion [5], information about the disease [6–8], coupled contagions of fear and disease [3,9] or game-theoretic analyses [10,11]. These approaches all represent different potential dynamic processes for social behaviour spread. Sparse empirical study to date is inconclusive in evaluating which of these formulations most appropriately represents features of real social behaviour change, or if as-yet-unexplored alternative approaches are needed [12–17]. Conceptually, existing formulations are generally limited to discrete behavioural states that update according to pairwise interactions among individual agents (at one extreme) or global states of the system (at the other extreme) [18–26]. Below, we present an alternative approach to modelling social spread of adaptive behaviour during epidemics which relaxes these assumptions—allowing behavioural dynamics to be shaped continuously by both local agent interactions and their broader social context. The mechanism we propose has already seen extensive uptake in other areas of health behaviour, which have lent some direct and indirect empirical support to our representation, and we will demonstrate that our approach offers improved flexibility and expressivity over other common approaches.

The central question addressed by our approach is: suppose an individual is considering whether to socially distance during an epidemic; what influences this decision? In the most common extant paradigm among epidemic models, an individual needs one or a pre-defined multiple of one exposure(s) to a behaviour of interest before adopting it, known as ‘simple’ and ‘complex’ contagions, respectively. Upon reaching the exposure threshold, behaviour tips with fixed probability between two states (e.g. actively self-isolating or not). Prior work proposed a ‘coupled contagion’ model [3], in which the disease, and fear of the disease both spread within a social system. Individuals could become ‘fearful’ from interacting with either an agent with the disease (the sick) or an agent with fear (the scared) and update their behaviour in response: either removing themselves from circulation or fleeing the interaction. Their results demonstrate that including adaptive behavioural responses can recover empirical phenomena observed during disease outbreaks: namely multiple waves of infection (when sequestered agents returned to circulation) and the acceleration of epidemics due to evacuation (which spreads the disease to the rest of the population). This modelling framework has been adopted and extended to numerous use cases [9,27,28].

In these previous works, behaviour and disease are seen as interacting contagions. They therefore follow the usual assumptions of contagion models. Individuals have one of a few discrete states—with at least one active and one inactive states such as sick or susceptible, informed or not, afraid or not—and the contagion spreads from individuals in one state to those in the other. This approach is mathematically convenient as both behaviour and disease can be tracked using similar mathematical or computational approaches. However, one may interpret this approach as a reductionist view of behaviour where individuals passively *adopt* (transition to) the state of their neighbours with fixed probability rather than *adapt* upstream elements such as information, perceptions, beliefs and opinions that affect decisions to adopt behaviours, with adaptation of decision components occurring continuously through social and epidemic dynamics. Importantly, adjusting the probability that a single agent changes their state is also only possible via an individual-based model. With this formulation, and unlike previous work on adoption of preventative behaviour, our model does not require that agents have a probabilistic awareness of their neighbour’s epidemiological state [29] and/or for them to compute global disease risk [30].

In our proposed alternative formulation, individuals instead ‘poll’ or ‘sense’ the full set of their friends, contacts or co-workers regarding *their* likelihoods of adopting the behaviour. Individuals then aggregate this information into an overall social composite and then move their own likelihood of behaviour in proportional response to this ‘social signal’. This formulation is neither pairwise (instead involving an aggregation mechanism that interacts information from across the entire set of social contacts) nor binary (rather than a fixed threshold at which behaviour tips from ‘no’ to ‘yes’, probabilistic shadings can vary smoothly across time and social space). Below, we explore a new paradigm for modelling adaptive social behaviour in epidemic models via this likelihood-updating mechanism, which we refer to hereafter as ‘social risk appraisal’ (SRA). Our specific initial conceptualization of SRA is built on the ‘follow the average’ mechanism, which is rooted in social conformity theory and has been widely utilized elsewhere in population health, notably as applied to the spread of obesity [25,31–36]. In particular, building on such theory allows our model to reflect active mechanisms of social learning rather than passive adoption of behaviour [37].

We argue that such a mechanism is needed because it can generatively capture temporal shifts in the distribution of an adopted behaviour. Most social behaviours can and do change over time, whether via cultural trends or the discovery of novel information. During the COVID-19 pandemic, extensive empirical work has shown shifting attitudes regarding the acceptability of non-pharmaceutical interventions [38,39], including social distancing, mask wearing, school closure, as well as the likelihood of vaccination [40]. As we will show, standard simple or complex contagion approaches may be insufficiently expressive or even misleading when the distribution of these behaviours changes over time.

Our results demonstrate that the SRA paradigm has important potential implications for modelling both the adoption of social behaviour in epidemic models and the subsequent impact on disease spread dynamics. We will show that disease spread outcomes under SRA often differ markedly from those generated using the canonical simple and complex contagion mechanisms for socially adaptive behaviour, thus motivating and informing further empirical research to identify which mechanism or mechanisms is likely operating in any given situation [1,4]. Uncovering why and how these differences in disease spread projections emerge shows not only their impact on potential policy or containment options but also different patterns of disease risk in the population, with potential equity implications. Our results also identify a key set of potentially measurable pre-conditions that could determine dynamics during an epidemic (the distribution across network structures of risk predispositions). Finally, we demonstrate that the SRA approach is highly flexible, subsuming existing paradigms and extending to a wide range of expressions including reversal of adaptive behaviours previously adopted by individuals. Our results are robust across a range of network structures, disease transmission probabilities and patterns of clustering in behaviour.

## 2. Results

The point of departure for our model is the coupled contagion process described in [3]. We first extend this model by mapping the agents onto a transmission network, allowing for direct comparison of coupled contagion with a standard alternative process (complex contagion of fear), and then implement as a third novel alternative the process of SRA in which agents update their likelihood of self-isolation (hereafter, ‘hiding’) towards the mean of their contacts. In SRA, rather than fear transmission being governed by a model parameter as in the original coupled contagion model, agents have an initial fear belief upon model instantiation, representing their probability of hiding if exposed to fear. Direct comparisons of the dynamics resulting from SRA and those from the coupled (simple) contagion and complex contagion alternatives are presented below.

### 2.1. Social risk appraisal dampens disease spread when initial belief distributions induce fear amplification

Consider a scenario where agents’ initial fear beliefs are distributed in the population such that most agents have a low fear belief (near 0), but a few have much higher ones. One such configuration is initial fear beliefs following a beta distribution:


(2.1) $$\begin{array}{c} \text{Fear belief}:\;\beta_{f}\;∼\;Beta\left(0.05,\;0.95\right). \end{array}$$

The beta distribution has support on [0,1], which we can model as the probability of a fear-exposed agent hiding. From basic probability, we also know the distribution’s third moment (skewness) is positive. When fear spreads via SRA and agents update their fear belief, the average fear in the population increases over time and eventually converges, which is well known when using a follow the average type mechanism [31]. We refer to such a scenario as ‘socially driven fear amplification’. The average fear belief upon model instantiation is about 0.05, and with step sizes of 5 × 10^–4^ under SRA, increases to about 0.1 over the course of 150 model timesteps.

**Figure 1.**
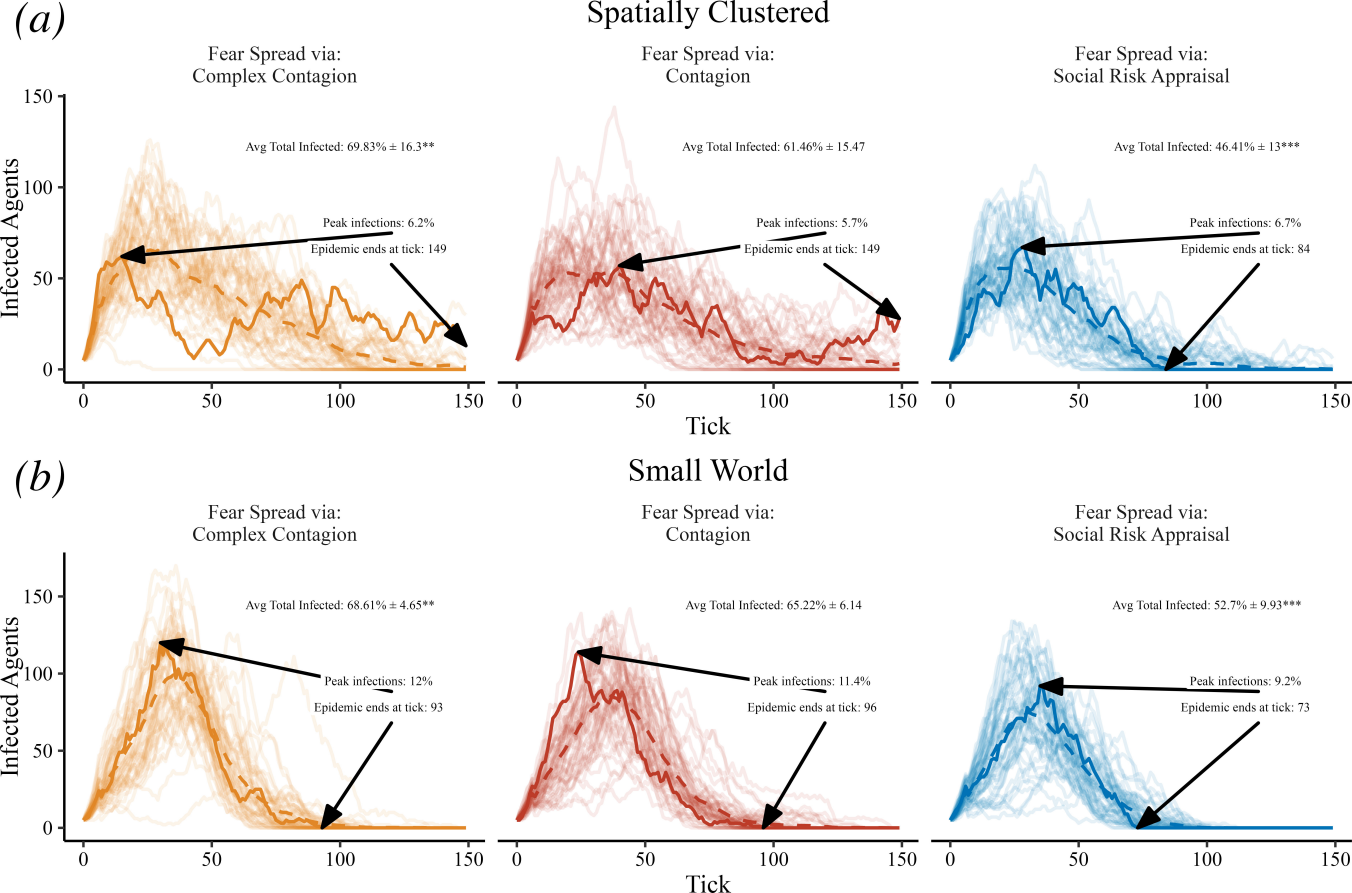
Socially driven fear amplification. Trace lines show the count of infected agents over time, and we highlight the unique model run leading to the 25th ranked (of 50) level of cumulative infections. Text labels indicate the average total number of agents infected during a model run with one standard deviation and a hypothesis test of the mean difference compared to simple contagion, the maximum number of agents infected at one time and the epidemic duration. Using a positively skewed distribution for fear beliefs leads to the average fear belief increasing over time when fear spreads via SRA. In this case, when comparing to fear spreading via simple contagion, SRA dampens epidemic spread. Resulting *p*-values for hypothesis tests are indicated with *p* < 0.001 (***), *p* < 0.01 (**), *p* < 0.05 (*) and *p* < 0.1 (.).

In figure 1, we visualize the results of socially driven fear amplification under SRA with simple and complex contagion mechanisms as comparisons. The incremental effect of allowing agents to update their fear beliefs via SRA in this case dampens the epidemic spread, on both a spatially clustered and a small world network. Complex contagion, with a threshold of two contacts to adopt the behaviour, on the other hand, leads to larger rates of infection. Dampened disease spread under SRA is also robust across multiple disease transmission rates, as shown in electronic supplementary material, figures S1 and S2. We show 50 replicate runs of each behavioural mechanism and parameter set, conducted with different random seeds. As representative run, we highlight the 25th ranked simulation with respect to cumulative infections (approximately the median) in order to show the nonlinear dynamics at play *within* a model run and also show the mean time series as a dashed line, offering multiple views of stochastic variability, consistent with standard best practice [41]. The same sets of random seeds are used consistently across experiments.

### 2.2. Social risk appraisal increases disease spread when initial belief distributions induce fear extinction

Knowing that SRA can dampen epidemic spread, can the same mechanism also induce the inverse behaviour? Consider a different initial fear belief distribution such that


(2.2) $$\begin{array}{c} \beta_{f}∼\max\left(N\left(0.05,0.05^{2}\right),0.01\right). \end{array}$$

For a normal distribution, the average fear belief will converge to the mean of the normal distribution due to symmetry. However, we make two small changes to the fear belief distribution: we floor the distribution at 0.01, and for agents with a fear belief of 0.01, give them a small step size (in this case, 10^−6^) with probability 0.25. This corresponds to about 50 of 1000 agents being ‘anchors’ who will change their fear beliefs more slowly than other agents. As a result, the average fear belief will *decrease* over time, leading to fewer agents hiding. We refer to such a scenario as ‘socially driven fear extinction’. When the non-anchor fear belief step size is 5 × 10^–3^, the average fear belief at the end of 150 timesteps is about 0.01. We argue that this distribution mirrors observed social behaviours in the population, where those holding a more extreme position (such as a low probability of hiding if exposed to fear) may be less likely to conform to a social norm [42]. We emphasize here that by imbuing some agents with smaller step sizes, we are still working with the parameters of our model, and that such an approach can generatively induce a decrease in the mean fear belief of the population.

**Figure 2.**
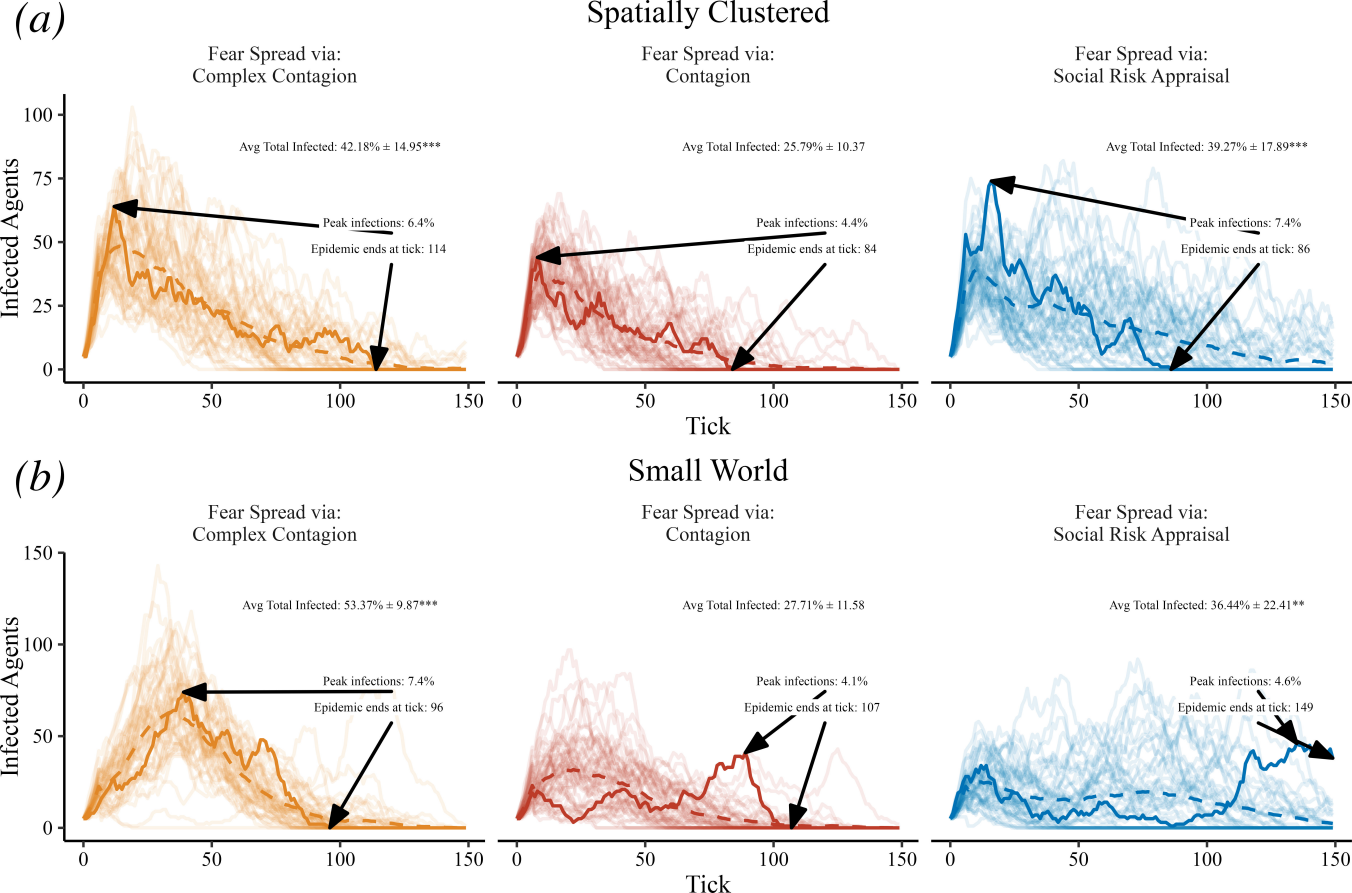
Socially driven fear extinction. Trace lines show the count of infected agents over time, and we highlight the unique model run leading to the 25th ranked (of 50) level of cumulative infections. Text labels indicate the average total number of agents infected during a model run with one standard deviation and a hypothesis test of the mean difference compared to simple contagion, the maximum number of agents infected at one time and the epidemic duration. Using a truncated normal distribution for fear beliefs with several agents serving as anchors leads to the average fear belief decreasing over time when fear spreads via SRA. In this case, when comparing to fear spreading via simple contagion, SRA worsens epidemic spread. Resulting *p*-values for hypothesis tests are indicated with *p* < 0.001 (***), *p* < 0.01 (**), *p* < 0.05 (*) and *p* < 0.1 (.).

The socially driven fear extinction experiment is summarized in figure 2, using the same networks, the same disease infection rates, and same initial mean fear belief as the socially driven fear amplification experiment described previously (in figure 1). Again, we emphasize that the only difference between the panels is the inclusion of SRA, where agents can update their fear beliefs. As before, we see that spreading fear via complex contagion worsens disease spread compared to simple contagion, but now we also see that SRA worsens disease spread as well, due to the average fear belief decreasing and fewer agents hiding. This demonstrates the richness of the SRA mechanism, since it can either dampen or ignite disease spread in the model, depending on the initial (potentially measurable) belief distribution of fear. We also note that SRA leads to later, more explosive peaks than simple or complex contagion, because fear extinction takes time to manifest in the agent population. More specifically, and in the same theme as the original coupled contagion model, the fear extinction scenario leads to having relatively more agents hide earlier in a simulation than at later timesteps, where far fewer agents hide, allowing for a larger mass of susceptible agents who can be infected. These results are also robust across disease infection rates and network types as shown in electronic supplementary material, figures S3 and S4.

### 2.3. Social risk appraisal changes the profile of which agents are most at risk of being infected

**Figure 3.**
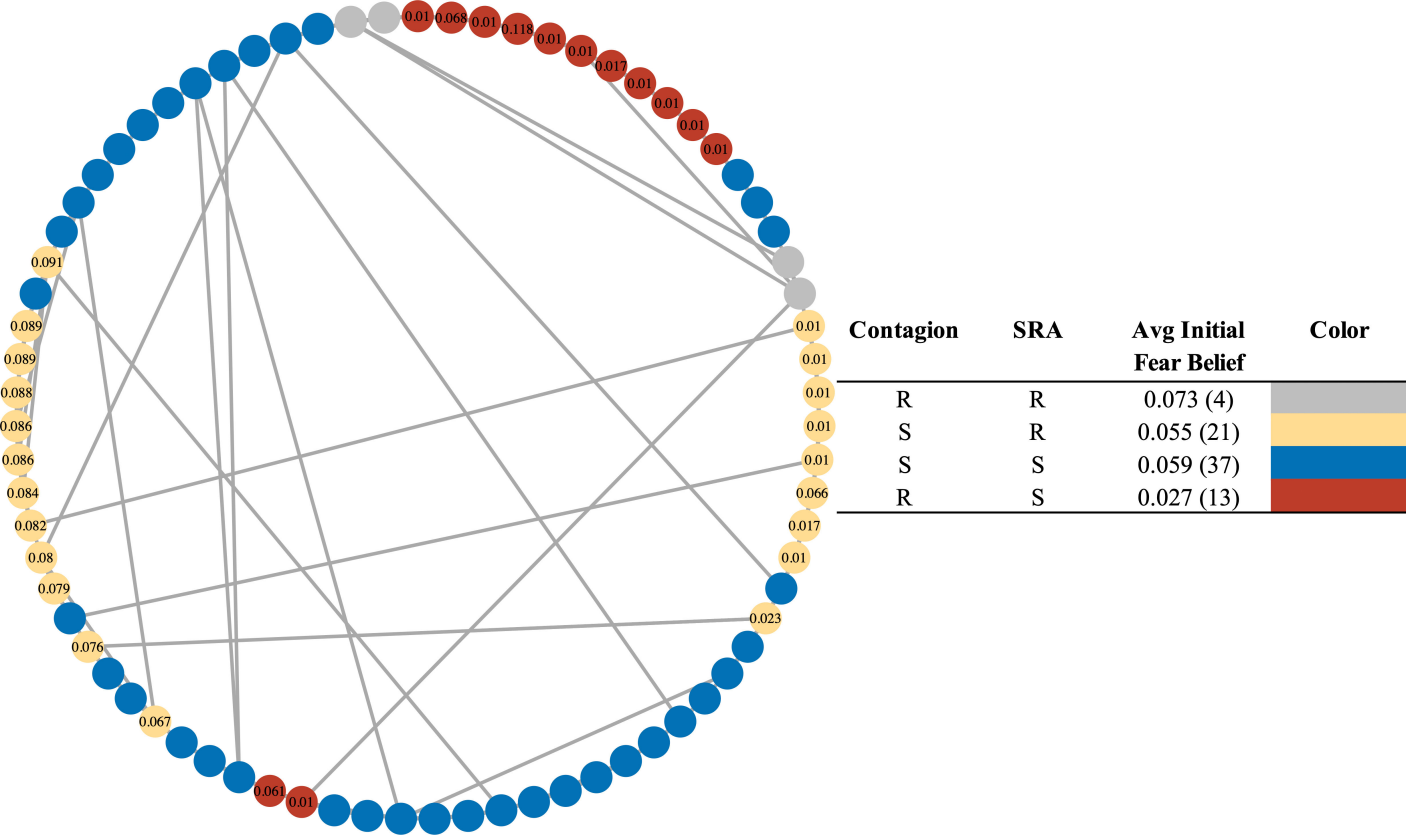
SRA’s effect on individual-level disease spread and risk of infection. Agents are coloured by their end disease state across two separate experiments run on the same random seed, but changing the fear spreading mechanism: one run with simple contagion, and the other via SRA. Blue agents are not infected in either experiment. Grey agents are infected in both experiments. Red agents are infected under simple contagion fear spread but not under SRA, and yellow agents are infected under SRA but not under simple contagion. Agents are labelled by their fear beliefs. Experiment was run with 75 agents, with 1 initial infected agent, on a clustered small-world network with an average node degree of 4, a rewiring probability of 0.15, and a node swapping probability of 0.1. In this case of this simulation, the SRA mechanism leads to the agents with the highest fear beliefs reducing theirs over time, leaving them more vulnerable than they were at model instantiation. In a scenario where higher initial fear beliefs may be characteristic of higher-risk populations (due to risk factors, or past experience), omission of the SRA mechanism may lead to a misrepresentation of which agents are most vulnerable.

Due to social influence under the SRA mechanism, an agent’s fear belief will change their level of risk of being infected during a simulation run. Thus, the configuration of the agents who are most at risk in the population under SRA may be different from in the case of fear spread via simple contagion, and this can lead to counterintuitive risk distributions in the population. Consider a simple schematic example of N=75 agents on a network clustered by their fear beliefs under socially driven fear extinction, the results of which are visualized in figure 3. To explore the mechanistic model differences induced by social conformity dynamics, we run two simulations on the same random seed, with the only difference being the change of the fear spreading mechanism (simple contagion versus SRA). We find that for this simulation, the population infected under SRA but not under simple contagion is characterized by *higher* initial fear beliefs (with an initial average fear belief of 0.055) than the population infected under contagion but not SRA (an initial average fear belief of 0.027). Although high initial fear beliefs would be expected to lead to more protective self-isolation and lower overall risk of infection, in this exploratory example, we find the reverse—agents starting with higher risk appraisal end up also at higher risk. This is due to the temporal shift of the fear beliefs induced by social influence which is characteristic of SRA, and further study is needed to understand general conditions under which this manifests and its impact on disease dynamics. If a more detailed, empirically grounded model was used to inform policy planning ahead of a disease outbreak, failing to account for this type of social dynamic could lead to a misrepresentation of the at-risk population, leading to a sub-optimal intervention, potentially worsening disease outcomes for the population at hand or sharpening inequities by worsening case rates among the most vulnerable populations. On the other hand, if adaptive behavioural social dynamics were explicitly modelled, a policy could account for how fast an intervention needed to be deployed, or where, or to whom, to protect at-risk populations whose ability (or likelihood) to self-isolate could change over time. This is especially crucial when the likelihood of engaging in a behaviour is clustered with respect to individual attributes (that is, displays homophily), which is a known feature of empirical social networks.

**Figure 4.**
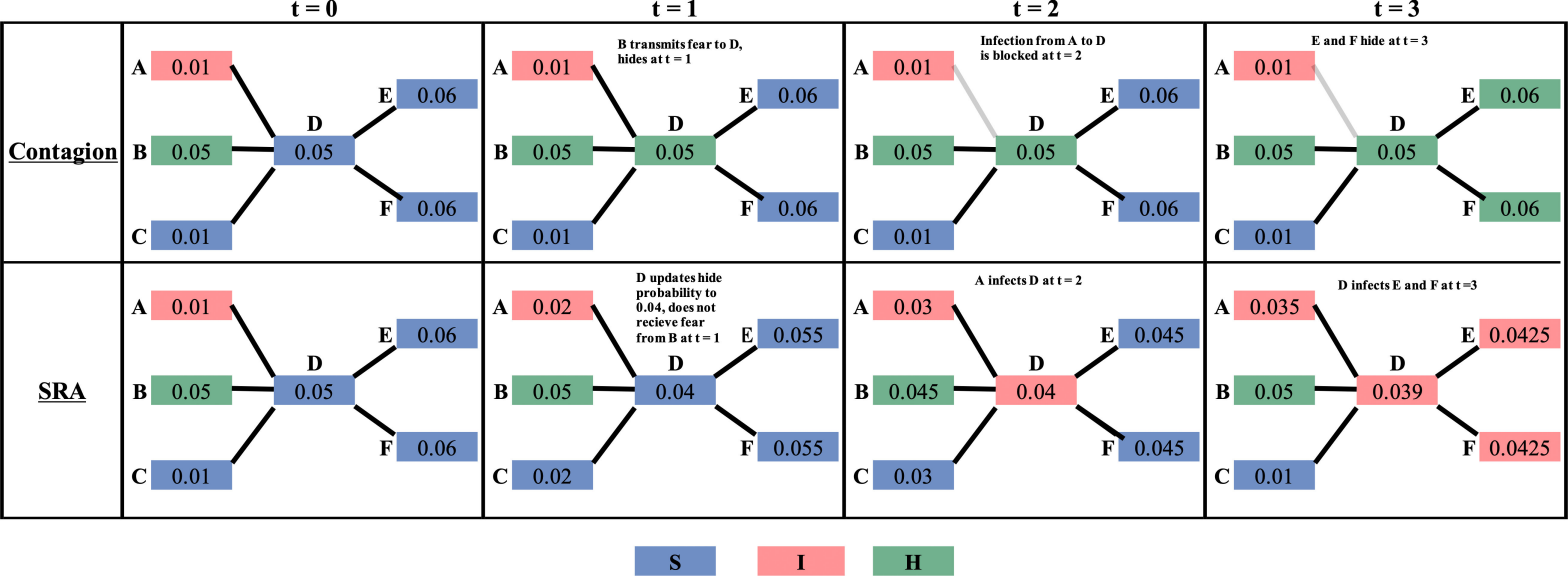
Five-agent single simulation stylistic example of fear transmission leading to change in risk profile over time. Agents, as boxes, are labelled with their fear beliefs. The top panels indicate fear spread via simple contagion, and the bottom panels indicate fear spread via SRA. Fear beliefs, disease state and fear state are all updated simultaneously for each agent at each timestep. Fear belief step sizes are fixed at 0.01 for all agents under SRA (or minimum distance to the mean fear belief of their contacts), chosen to allow fear belief updating within a short number of model timesteps. Assume that at timestep *t* = 1, agent D hides under the simple contagion case (with hiding probability 0.05), but does not under SRA (due to the decrease in fear belief to 0.04). Under SRA, agents E and F more vulnerable at time *t* = 3 due to D’s infection, as well as their decrease in hiding probabilities (0.06 at *t* = 0 and 0.0425 at *t* = 3) and subsequently are infected.

To build intuition for these dynamics, consider a second worked example with five agents, four timesteps and fear belief step sizes fixed at 0.01, which is displayed in figure 4. Under simple contagion, at *t* = 1, agent B transmits fear to agent D, who hides with probability 0.05 and is then blocked from infection with the pathogen from agent A. Agent D also subsequently transmits fear to agents E and F at time *t* = 3, who hide with probability 0.06. Under SRA at *t* = 0, agent D would update its fear belief towards the mean of its contacts, in this case decreasing to 0.04 (since the mean fear belief of its contacts is 0.039). Thus, at *t* = 1, D is less likely to hide under SRA than under simple contagion. In our stochastic transmission model, this could lead to A infecting D at *t* = 2, and then D infecting E and F at *t* = 3, who also decreased their fear beliefs in a similar manner. We emphasize here that these are only two realizations of a stochastic model but still enumerates explanations for how SRA can alter the profile of which agents are most at risk of infection.

### 2.4. Social risk appraisal is robust to clustered social behaviour

**Figure 5.**
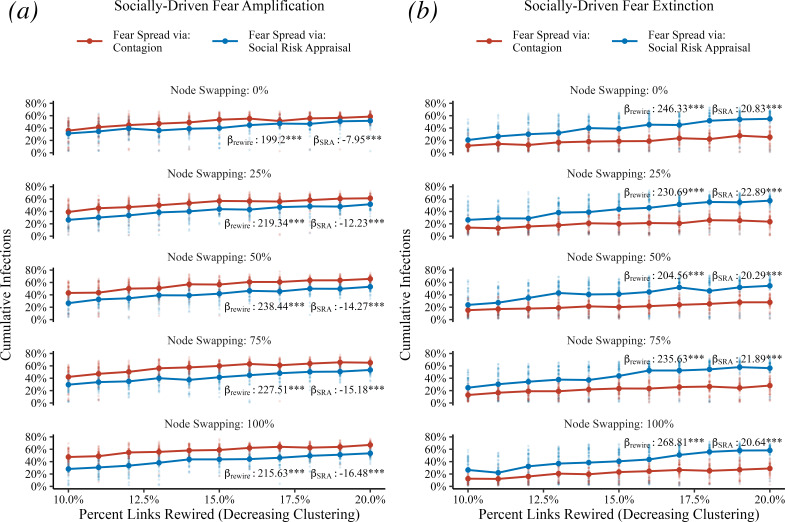
SRA’s effect on cumulative disease spread is robust across social behavioural clustering. (*a*) The results of socially driven fear amplification and (*b*) details of socially driven fear extinction. In each panel, the horizontal axis indicates the amount of random rewiring of links in the network (which decreases clustering in the network). The vertical axis represents the percent of agents infected at the end of a model run. Within each panel, individual plots show levels of node swapping on the ring lattice before neighbouring ties are created, controlling the correlation of agents’ fear beliefs among their neighbours. Panels also contain regressions for cumulative infection as a function of the experiment (SRA or contagion, $\beta_{\text{SRA}}$) while controlling for link rewiring ($\beta_{\text{rewire}}$). Coefficient estimates were all significant with *p* < 0.001. As a sensitivity we also ran regressions controlling for node swapping: under fear amplification $\beta_{\text{SRA}}$ = −13.22 with *p* < 0.001 and for fear extinction $\beta_{\text{SRA}}$ = 21.31 with *p* < 0.001.

A key feature of real social networks is the presence of homophily: that is, if we were observing the fear beliefs of agents in the real world, we would expect agents’ fear beliefs to be clustered together, rather than assigned randomly in the population in our initial experiments. We introduce a clustered small-world network that follows the same construction as the Watts–Strogatz network [43], but when agents are arranged into a ring lattice, they are instead first sorted by their fear probability and allowed to swap positions randomly before forming their connections. This allows agents with similar fear beliefs to be connected with one another, with sufficient heterogeneity such that an individual agent’s fear belief will change during a model run. We see that across a range of clustered networks that in the socially driven fear amplification and fear extinction experiments, our main results hold: under fear amplification, SRA dampens disease spread, and under fear extinction, SRA increases disease spread (figure 5).

### 2.5. Social risk appraisal can both reproduce key findings of alternatives and extend them

**Figure 6.**
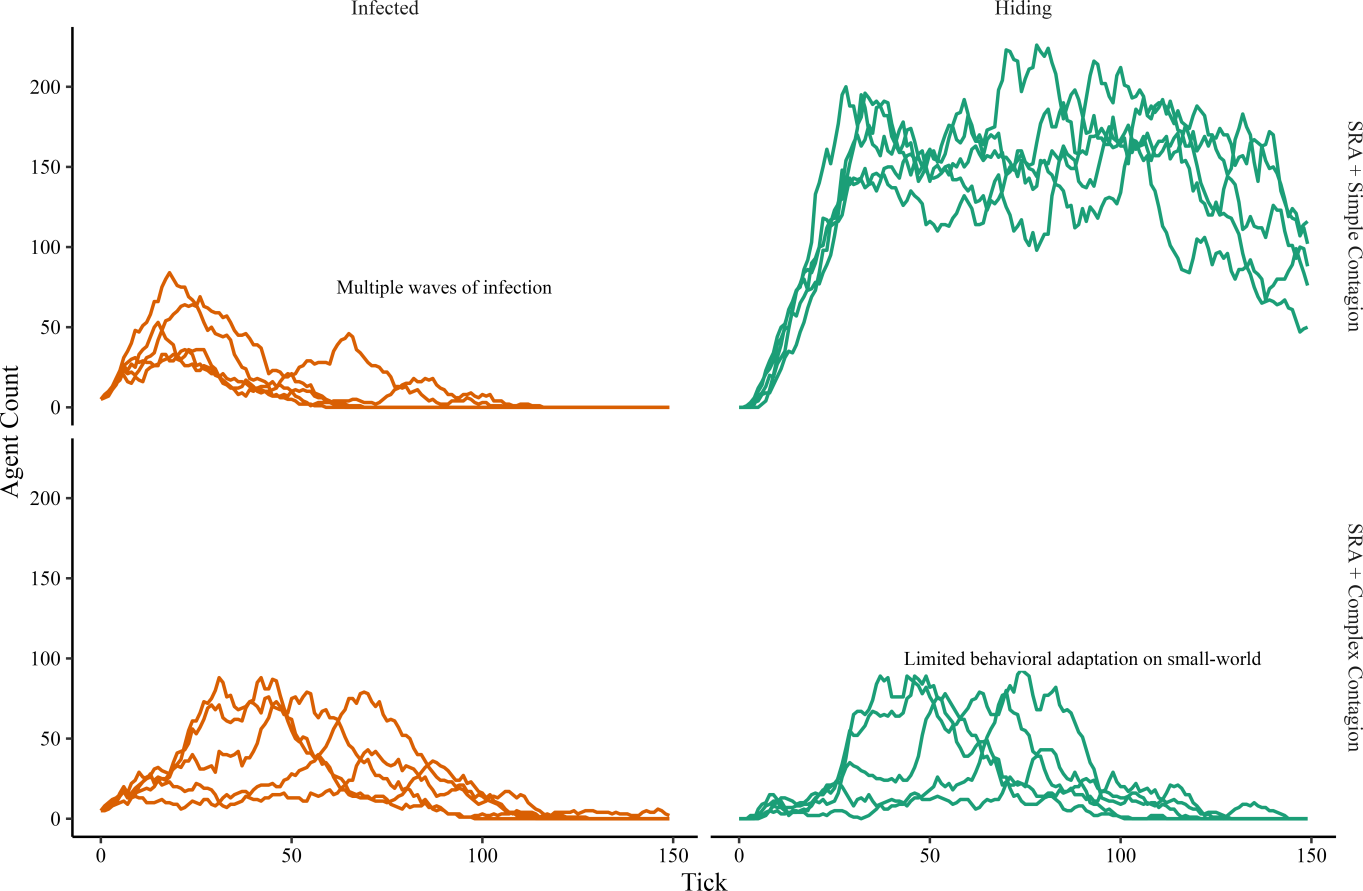
Recovering qualitative conclusions of canonical models. The left-hand panels show multiple waves of infection with the SRA framework, especially in the case where SRA is combined with simple contagion, consistent with [3]. The right-hand panels show how SRA and complex contagion can reduce behavioural adoption on small-world networks compared to simple contagion, consistent with [2]. Results are shown for a socially driven fear extinction experiment.

Other work has demonstrated several key contributions of incorporating coupled contagions and complex contagions. Our model, with the right parametrizations, can subsume the behaviour of these earlier works. In particular, we focus on two canonical findings in the coupled and complex contagion literature. The first is that coupled contagion can lead to multiple waves of infection: during the first wave, agents hide and remove themselves from circulation, and then upon returning before the epidemic has completely diminished, fuel a second wave of disease spread [3]. The second is that behavioural spread is limited on small-world networks [2]. Figure 6 summarizes these qualitative takeaways. We emphasize the generality of our modelling approach here: when layering SRA onto our other two fear-spreading mechanisms, we can still recover the main findings of other works under certain parametrizations.

Thus far, we have focused on SRA as a mechanism driving adoption of a behaviour when the likelihood of adopting the behaviour is heterogeneously distributed in the population. Our final analysis investigates the ramifications of allowing the *release* from the social behaviour (in this case, an agent removing themselves from hiding) to also depend on an agent’s fear belief.

Our formulation of the release from hiding departs from the canonical literature: most models assume either a fixed duration that each individual hides or individuals probabilistically returning to circulation, also with a fixed probability. Instead, we model the probability that a hiding agent continues to hide as


(2.3) $$\begin{array}{c} \mathrm{Pr}\left(continue\;to\;hide|hiding\right)=\exp\left(\frac{-1}{a*\beta_{f}\;}time\;hidden\right),\;a=150 \end{array}.$$

Under this functional form, higher fear belief agents are likely to hide for longer, and lower fear belief agents are more likely to hide for a shorter amount of time. The parameter a is calibrated such that the average hide time for an agent under simple contagion is t=4 days (electronic supplementary material, figure S5), consistent with our earlier experiments.

This form is also similar to a Rescorla–Wagner interpretation of fear decay [9]. The longer an agent is away from the fear exposure (which caused them to hide), the higher the likelihood they have in returning to circulation. However, we depart from Rescorla–Wagner in that we do not model fear decay as a non-cognitive behaviour, but rather as a socially driven cognitive one under SRA. In reality, the decision to socially distance and the decision to return to circulation may be governed by both cognitive and non-cognitive behavioural factors, and thus incorporating social influence sets the stage for future exploration to include both of these approaches.

**Figure 7.**
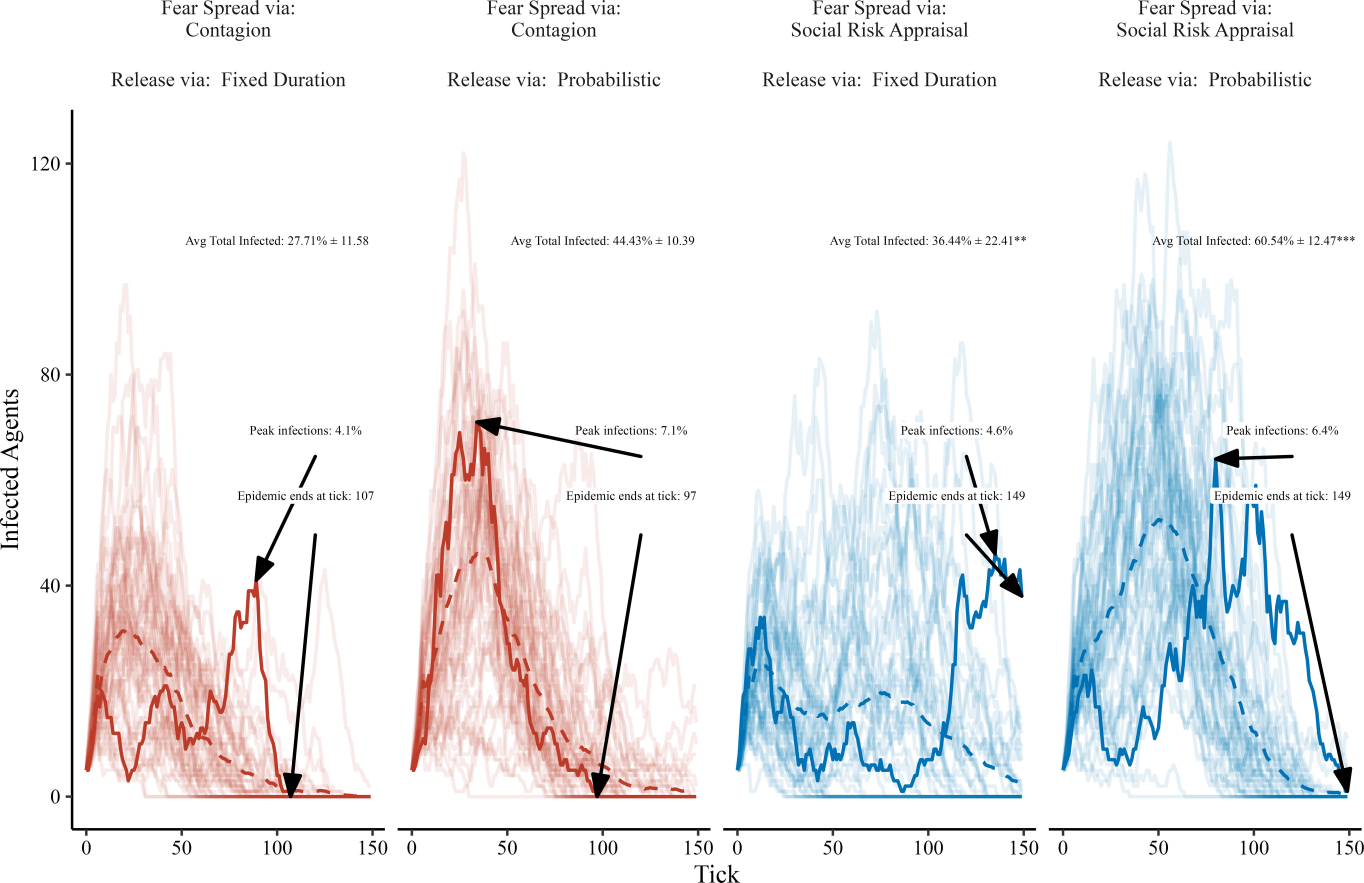
Socially driven fear extinction with probabilistic release. Simulations vary the fear spreading mechanism and the release mechanism. Trace lines show the count of infected agents over time, and we highlight the unique model run leading to the 25th ranked (of 50) level of cumulative infections. Text labels indicate the average total number of agents infected during a model run with one standard deviation and a hypothesis test of the mean difference compared to simple contagion, the maximum number of agents infected at one time and the epidemic duration. Allowing the release mechanism to be governed by SRA leads to increased overall disease spread compared to a fixed hiding duration. Resulting *p*-values for hypothesis tests are indicated with *p* < 0.001 (***), *p* < 0.01 (**), *p* < 0.05 (*) and *p* < 0.1 (.).

When we allow an agent’s decision to return to circulation to also include the SRA framework, we find that our earlier results still hold in the case of the socially driven fear extinction experiment on a small-world network, as summarized in figure 7. When comparing fear spreading mechanisms and release mechanisms, we see that SRA still leads to later, more explosive epidemic peaks and overall cumulative disease spread for both the fixed duration hiding as well as the probabilistic release. Notably, we also see that combining SRA for both the onset and release of hiding leads to the latest, highest peak across all other panels. The results are also robust across disease transmission rates, as shown in electronic supplementary material, figure S6.

## 3. Discussion

Our simulations underline the need to include appropriate and well-grounded socially adaptive behaviour in epidemiological models. Emerging from simple interaction rules, our SRA mechanism produces quite different dynamics from those of alternative formulations of adaptive self-isolation behaviour and can lead to widely different levels of disease spread depending on pre-conditions that are not yet well measured empirically (either dampening or worsening simulated epidemics as well as changing the distribution of risk in the population). In the case of a long-running epidemic such as COVID-19, this would be especially important, as perceptions of risk about infection have varied widely over geospatial context and time [39,44,45]. Detailed data on the rate of social conformity and the distribution of risk perception (and therefore protective self-isolation) would help one parametrize the SRA mechanism. Consider a scenario where a set of stakeholders wanted to understand the conditions under which disease would spread in a community and how to best plan epidemic response scenarios. Failing to account for social dynamics appropriately could result in overly optimistic or pessimistic predictions of disease spread and thus a potential mismatch between containment policy and true underlying epidemic dynamics.

We do not claim that our exact formulation of SRA is the only or even the optimal way to view social dynamics. Rather, we show that SRA is an important third potential formulation for how self-protective adaptive behaviour may spread in the population which differs markedly from existing alternatives of simple and complex contagions. SRA enjoys conceptual and theoretical advantages, and brings some empirical and conceptual support from other applications in population health. Agent-based models are nearly unlimited in the behaviours they can express. Given a large enough set of parameters, enough time and enough data, it is quite likely we could generate *any* set of epidemiological outputs in addition to the results we have shared above. However, in order to make our models tractable and understandable, modellers often prefer simple mechanisms, grounded in theory and data, that can express a wide range of behaviours [41]. Though the mechanism of action in SRA is simple, the framework is generic, governed by the initial distribution of the risk assessment and the manner in which it is updated; further study is needed to elucidate the conditions under which these dampen, worsen or otherwise shape epidemic spread. We also anticipate that these questions can be investigated in future modelling work, as general conditions in high-dimensional complex systems are challenging to express in closed form, and the careful design and exploration of these dynamics depend on the modelling problem at hand [46]. We argue here that including social influence in contagion models via SRA opens the door to a rich set of model outputs and behaviours and should be considered as a viable mechanism to model relevant social behaviours in epidemic models, including self-isolation, mask-wearing, diagnostic testing and vaccination, both individually and in combination.

One area for further development that the SRA framework might facilitate builds on recent results from social learning models when direct learning is challenging since polling or sensing the behaviours of others or their likelihood of adopting a behaviour is not straightforward. In some cases, learning of health-related behaviour may be conditional on an agent’s health status (individuals might listen more to their healthy neighbours in some cases, but more to their infected neighbours in others). Interestingly, in the case of infectious diseases, an agent’s health is itself a collective artefact of their own behaviour and of their neighbour’s behaviours. Behavioural adaptation then becomes a collective intelligence task [47]. Future models could therefore combine SRA with recent developments in social learning through noisy collective performance [48].

Recent work in epidemiology has advocated more broadly for inclusion of richer social and behavioural dynamics via agent-based models [1]. One of the primary recommendations was to build a community of practice among social scientists, risk communication and community engagement practitioners, and epidemiologists. Our modelling approach is well posed to engage with this community, where social scientists and epidemiological modellers could collaborate and explore how including SRA may affect disease dynamics across a wide range of social behaviours and epidemiological scenarios. Our work could be extended in several ways. As currently implemented, our model uses the same network for disease and fear transmission, but our model could easily be extended to accommodate a multi-layer network, capturing the highly nonlinear (i.e. scale-free) characteristics of information spread on the World Wide Web [49]. Additionally, while not explored here, we recognize there is a vast literature of decision making and behavioural adoption, which are likely a function of an individual’s geographic context, economic status and lived experience, any of which could be incorporated into our framework to continue exploring the myriad factors that shape social behaviour and its impact on disease spread. Decisions about whether and how to include SRA in epidemiologic models can—and, we believe, should—be supported with future empirical studies of behavioural adaptation. Although such research presents myriad daunting methodological challenges (adaptation processes are not directly observable and people may not be able to fully articulate the factors that drive their actions), the results we present here provide additional motivation for future research efforts in this direction.

## 4. Methods

### 4.1. Overview

Our model extends the well-known coupled contagion agent-based model [3]. We review the core dynamics of that model below, focusing on the aspects where our model differs. Primarily, we move the model environment from a lattice to a network, imbue agents with their own likelihood of adopting the protective behaviour and allow for differential mechanisms of behavioural adoption.

### 4.2. Instantiation and dynamics

Our model consists of *n* = 1000 agents, who have two governing ‘states’ which define their behaviour. Agents have a *pathogen* (or disease) state corresponding to the classic SIR model [50–52], where they are Susceptible (S) to, Infected (I) with, or Recovered (R) from the pathogen. Simultaneously, *fear* of the disease may also spread, and agents may remove themselves from circulation for a short of time, upon which they cannot be infected with the pathogen. Agents may be infected with either or both the pathogen and fear.

Agents may contract fear by being connected to a pathogen-infected agent or a fear-infected agent. Each agent has a ‘fear belief’ representing the probability that they will hide when exposed to a fearful agent. Our model includes three fear-spreading mechanisms. The first is identical to the coupled contagion model, where fear spreads via simple contagion. Specifically, define for agent *i*:


(4.1) $$\text{Pathogen state: }P_{i}\in \left\{S_{P},I_{P},R_{P}\right\},$$

(4.2) $$\text{Fear state: }F_{i}\in \left\{S_{F},I_{F}\right\},$$

and a list of their network contacts $C_{i}$ as well as their pathogen-infected contacts $PI_{i}:\left\{j\in C_{i}|\;P_{j}=I_{P}\right\}.$ At each timestep,$\;P_{i}$ transitions from $S_{P}$ to $I_{P}$ if


(4.3) $$\begin{array}{c} \sum_{j\in PI_{i}}I_{X<\beta_{P}}>0 \end{array},$$

where $I_{X<\beta_{P}}$ represents the indicator function for the event $X<\beta_{P}$ where X is a uniform random variable on [0,1] and $\beta_{P}$ is the disease infection rate for the model. Pathogen-infected agents may also spread fear to their contacts in the same manner, where $F_{i}$ transitions from $S_{F}$ to $I_{F}$ if


(4.4) $$\begin{array}{c} \sum_{j\in PI_{i}}I_{X<\beta_{f_{i}}}>0 \end{array},$$

where $\beta_{f_{i}}$ represents an individual agent’s fear belief. Fear-infected agents may also spread fear to their contacts. In this case, define for agent *i* their set of fear-infected contacts $FI_{i}:\;\left\{j\in C_{i}|\;F_{j}=I_{F}\right\}.$ $F_{i}$ transitions from $S_{F}$ to $I_{F}$ and then removes themselves from circulation (i.e. cannot contract the pathogen) if


(4.5) $$\begin{array}{c} \sum_{j\in FI_{i}}I_{X<\beta_{f_{i}}}>0 \end{array}.$$

**Figure 8.**
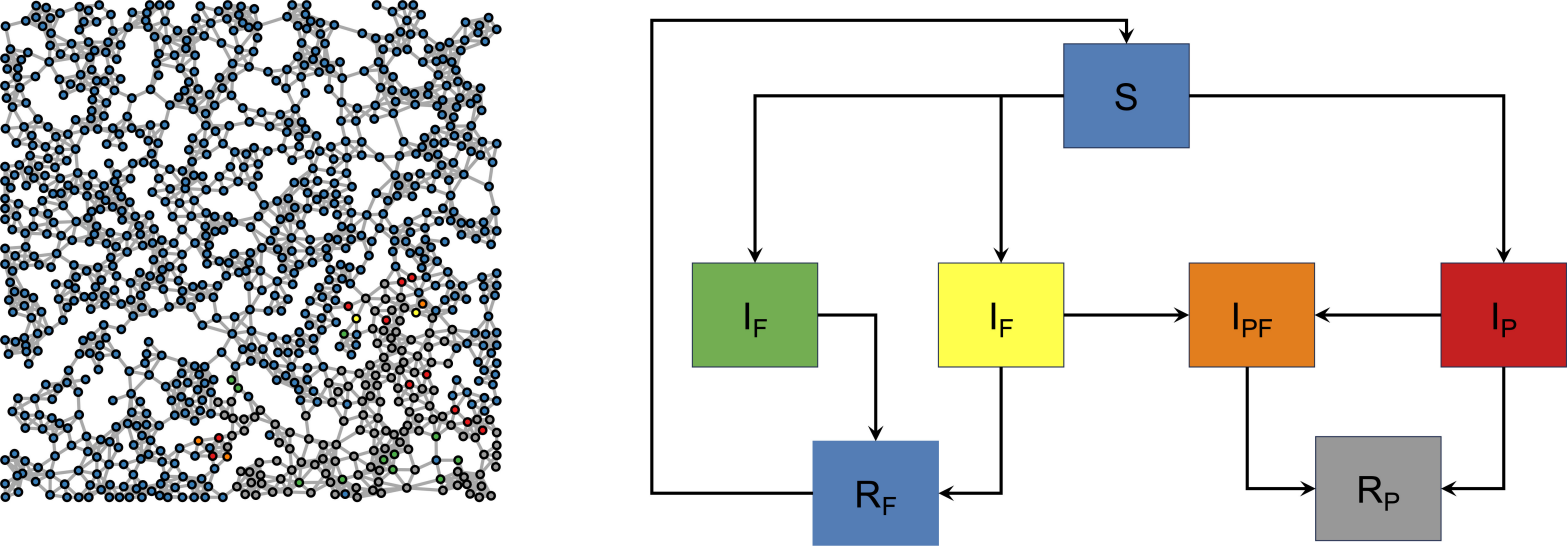
Coupled contagion on a network. Colours indicate the disease and fear states of agents, who all begin uninfected with the pathogen or fear (blue). Agents may be infected with the pathogen (red), infected with the pathogen and with fear (orange) infected with fear from a fearful contact (green), infected with fear from a sick contact (yellow) or may be recovered from the pathogen (grey). Subscripts F and P denote fear and pathogen, respectively. Pathogen and fear states are the same as for the original coupled contagion model [3].

A visual overview of the standard (simple) coupled contagion model is given in figure 8. The left-hand panel of the figure visualizes a sample network, in this case a ‘spatially clustered’ version [53] midway through a model run, and the right-hand panel enumerates the disease and behaviour transmission pathways. The network also demonstrates the inherent nonlinear dynamics at play, where some regions (in this case, the lower right-hand side) have more fear spread than disease spread, and some regions where the pathogen dominates (such as in the centre right-hand side).

The second mechanism is fear spreading via complex contagion, where an agent must have multiple exposures from fear-infected agents to hide. In this case, when agent *i* interacts with their fear-infected contacts, the size of ${FI}_{i}$ must be at least as large as the complex contagion threshold (in our case, requiring two fear-infected contacts) to hide.

Our third fear mechanism is SRA: fear still spreads via simple or complex contagion, but after each timestep of the model, agents update their fear belief by a small step size in the direction of the average fear belief of their contacts. Specifically, each agent *i* updates their fear belief $\beta_{f_{i}}$ in the direction of


(4.6) $$\begin{array}{c} \text{s}\text{i}\text{g}\text{n}\left(\frac{1}{\left|C_{i}\right|}\sum_{j\in C_{i}}\beta_{f_{j}}-\beta_{f_{i}}\right) \end{array}$$

and of magnitude


(4.7) $$\begin{array}{c} \min\left\{\text{abs}\left(\frac{1}{\left|C_{i}\right|}\sum_{j\in C_{i}}\beta_{f_{j}}-\beta_{f_{i}}\right),\;\text{step\_size}\right\} \end{array},$$

where step_size is a model parameter. This mechanism is based on a ‘follow the average’ approach, which has been applied to the spread of obesity in a social system [31,32]. SRA allows us to capture two crucial aspects of social behaviour: individuals updating their likelihood of adopting a behaviour and social conformity.

Our three fear-spreading mechanisms may be viewed as special cases of a more general framework. The complex contagion case subsumes the simple contagion case when the exposure threshold is set to 1, and the SRA mechanism subsumes the simple contagion case when all agents have the same initial fear belief (and therefore agents would not update their beliefs).

### 4.3. Modelling the release from fear

In the majority of our experiments, agents hide for a fixed duration of t=4 timesteps. Agents who have not been infected with the disease may hide any amount of times when exposed to a fearful contact. We also allow for a separate release mechanism, where the probability of a hiding agent continuing to hide is a damped exponential function of the agent’s fear belief, as described in equation (2.3). For our socially driven fear extinction experiment with probabilistic removal from hiding, we calibrate our release mechanism such that isolating agents hide for four timesteps. The results of the probabilistic release mechanism calibration are given in electronic supplementary material, figure S5.

### 4.4. Transmission networks

Transmission of both fear and disease takes place over a network. In the present work, we include two types of canonical networks: spatially clustered [53], small-world [43], as well as a third, customized network. Our third network is a variant of the small-world model, but allows for agents to be clustered by their fear belief. Specifically, in the first step of the Watts–Strogatz algorithm, we sort agents by their fear probability, thus allowing agents with similar fear probabilities to be close to one another. Before connecting agents to their neighbours, we allow for each agent to randomly swap places in the ring with another agent with probability $p_{swap}$. The Watts–Strogatz algorithm then proceeds as normal.

### 4.5. Model timestep

At each timestep of the model, agents perform the following actions:

Update disease state: disease-infected agents may infect susceptible agents with some probability. Disease-infected agents may also transmit fear to susceptible agents with probability corresponding to the receiving agent’s fear belief. These fear-infected agents do not hide but can spread fear. Disease-infected agents recover from the disease after a fixed number of timesteps and move to the recovered state.

Update fear state: fear-infected agents may transmit fear to fear-susceptible agents. These fear-susceptible agents hide with probability corresponding to their fear belief. While hiding, agents cannot be infected with the disease. Depending on the release mechanism, agents either hide for a fixed number of timesteps or return to circulation with probability corresponding to their fear belief and amount of time hidden.

Update fear belief: under SRA, agents will update their fear belief by a small step size in the direction of the mean fear belief of their contacts.

### 4.6. Implementation and experiments

Our model is programmed in NetLogo [54] v. 6.2.2. Each of our experiments runs for 150 discrete timesteps. The constant (non-varying across experiments) parameters of the model are given in table 1.

**Table 1.** Constant model parameters for experiments.

parameter	description	value
initial infected	number of agents infected at model start	5
disease infection rate $\beta_{p}$	pathogen transmission probability	0.09 for spatially clustered network; 0.06 for small-world network
fear infection rate $\beta_{f}$	mean fear transmission probability upon model instantiation	0.05
step increment	fear probability update size under SRA	0.0005 for socially driven fear amplification; 0.005 for socially driven fear extinction
hide duration	number of ticks an agent hides	4
network type		spatially clustered; small world; clustered small world
average node degree	mean number of connections for each agent	6
number of agents		1000
infection duration	number of ticks an agent is infected and infectious for	6

### 4.7. Data analysis

In figures 1, 2, 5 and 7 each experiment contains 50 replicates. For figures 1, 2 and 7 we sort the model runs by cumulative infections at the last model timestep and highlight the model run leading to the 25th ranked level of cumulative infections (approximately the median model run). Linear regressions and Welch’s *t*-tests were conducted using R 4.4.1 [55]. Resulting *p*-values for hypothesis tests are indicated with *p* < 0.001 (***), *p* < 0.01 (**), *p* < 0.05 (*) and *p* < 0.1 (.).

### 4.8. Sensitivity analysis

As a sensitivity analysis, we include results from a series of experiments where hiding agents cannot spread fear while they are hiding. Our main findings that SRA dampens disease spread under socially driven fear amplification and worsens disease spread under socially driven fear extinction also hold under this configuration. These results are available in electronic supplementary material, figures S7–S12.

## Data Availability

All simulation data and code used in this study are available on GitHub at https://github.com/davidogara/Social-Risk-Appraisal/ and are archived via Zenodo [56]. Electronic supplementary material is available online [57].
